# Mechanical force promotes tissue and molecular changes in adipose tissue regeneration post-transplantation

**DOI:** 10.3389/fcell.2024.1472575

**Published:** 2024-09-18

**Authors:** Yuan Ye, Jian Ma, Bing-yang Guo, Xiong-jie Li, Kui-kui Hu, Mei-jun Tan, Liang Zhang

**Affiliations:** ^1^ Department of Plastic and Cosmetic Surgery, Guangdong Women and Children Hospital, Guangzhou, China; ^2^ Translational Medicine Center, Guangdong Women and Children Hospital, Guangzhou, China

**Keywords:** mechanical shear force, stromal vascular fraction gel, coleman fat, tissue regeneration, transcriptome

## Abstract

**Introduction:**

Fat grafting often yields inconsistent and suboptimal results, necessitating improved fat processing techniques. A stromal vascular fraction (SVF) gel created using mechanical emulsification demonstrates superior retention rates to conventional Coleman fat grafts.

**Methods:**

This study investigated the mechanisms at play by transplanting fat aspirates from liposuction patients—either processed as Coleman fat grafts or further refined into an SVF gel via mechanical shear force—onto the backs of nude mice.

**Results:**

The retention rate of the SVF gel after transplantation surpassed that observed for Coleman fat. Hematoxylin and eosin (HE) staining and immunofluorescence results demonstrated that the SVF gel group could form new adipose tissue characterized by well-organized mature fat structures. Mechanical shear force application induced increased mesenchymal stem cell abundance. Rather than merely surviving regeneration, fat was regenerated after transplantation, and the regenerated cells were mainly from mice, which was supported by microarray analysis. RNA-seq highlighted 601 genes expressed between SVF gel and Coleman fat groups, with 164 genes upregulated (cell cycle processes), and 437 genes downregulated (lipid metabolism).

**Discussion:**

The application of mechanical shear force reduces the risk of complications and fosters cell proliferation and division, thereby enhancing the retention and regeneration of transplanted fat.

## 1 Introduction

Fat transplantation is increasingly being recognized for its importance in tissue repair and reconstruction (Cohen et al., 2017; Maximiliano et al., 2020; Tanwar et al., 2022), although its long-term retention is notoriously unpredictable, hindering its broader clinical adoption (Vyas et al., 2020). The success of fat transplantation hinges on various factors, primarily the method of fat treatment prior to transplantation (Gentile et al., 2019; Oranges et al., 2019; Ruan et al., 2019). Extensive research has been devoted to developing treatments or design strategies that enhance the post-transplantation retention rate of fat. Initial studies attributed the key to retaining fat to the preservation of adipocytes’ structural integrity and activity, a concept known as the cell survival theory (Coleman, 2006a). However, recent research has shifted the understanding of adipogenesis towards “the host cell replacement theory,” suggesting that neonatal adipocytes largely arise through the differentiation of regeneration-associated cells. This paradigm shift underscores the importance of exploring innovative approaches to improve fat graft longevity by focusing on regeneration-related cellular mechanisms (Coleman, 2006b; Dong et al., 2015; Kato et al., 2014; Liu et al., 2023).

The key to enhancing graft regeneration is acquiring more regeneration-associated cells, particularly adipose tissue-derived mesenchymal stromal cells (ASCs). Traditional methods for cultivating ASCs in large quantities involve using collagenase to digest fat tissue, a technique unsuitable for human application (Gentile et al., 2019). In 2017, Yao et al. (2017) developed an innovative approach, creating a gelatinous stromal vascular fraction (SVF) fat by mechanically shearing Coleman fat grafts. This SVF gel, rich in regeneration-associated cells like ASCs, shows considerable promise for cell therapy. Unlike traditional methods, which rely on enzymatic digestion with agents like collagenase that pose various risks, such as potential allergic reactions and cell contamination during extended lab culture, SVF gel technology utilizes a purely physical method—mechanical shearing—making it a safer alternative. This method effectively destroys mature fat cells while preserving the regenerative and differentiation capabilities of stem cells. However, the exact mechanisms by which shear force application enhances cell regeneration in SVF gels remain to be fully elucidated.

In our previous research, we utilized flow cytometry to compare cell subsets under varying shear forces, elucidating cellular-level changes and demonstrating an increase in regeneration-associated cells. We discovered that the SVF gel created through mechanical shearing and devoid of enzymatic digestion achieves efficient and stable volume enhancement and significantly enriches the population expressing mesenchymal stem cell markers compared with traditional lipotransfer techniques (Yao et al., 2017). This suggests that SVF gels have distinct advantages over conventional Coleman fat in clinical transplantation, including the reduction of mature fat cells and an increase in cells with high regenerative potential. Various studies, including ours, have used immunofluorescence staining to demonstrate that new adipocytes originate from host-derived adipose-related cells (Doi et al., 2015; Dong et al., 2015; Yao et al., 2017; Ye et al., 2021). Nonetheless, a major challenge in fat transplantation research lies in its reliance on imaging observations without comprehensive molecular analysis. Therefore, exploring the mechanisms underlying adipose regeneration induced by mechanical isolation through molecular biology techniques becomes essential for advancing the field (Shahriar et al., 2024).

To bridge this knowledge gap, we performed a transcriptome analysis of adipose tissue from Coleman fat and SVF gel samples transplanted into nude mice. This approach utilizes microarrays and RNA-seq as powerful tools to dissect the molecular mechanisms underlying the observed differences in fat regeneration post-transplantation. Gene expression exhibits robust dynamics and can serve as a real-time indicator of molecular changes in response to external stimuli within cells. Currently, microarray or RNA-seq techniques enable simultaneous detection of dynamic mRNA changes on a genome-wide scale. We used these methods to investigate the molecular events following the transplantation of mechanically processed SVF gel compared with unprocessed Coleman fat. We aimed to elucidate the mechanisms underlying adipose tissue regeneration facilitated by mechanical shear forces. Additionally, this research may unveil specific molecules that serve as promising targets to enhance the retention rate of transplanted fat through strategic pre-treatment, offering new directions to improve the efficacy of fat transplantation therapies.

## 2 Materials and methods

### 2.1 Fat sample acquisition and processing

**FIGURE 1:**
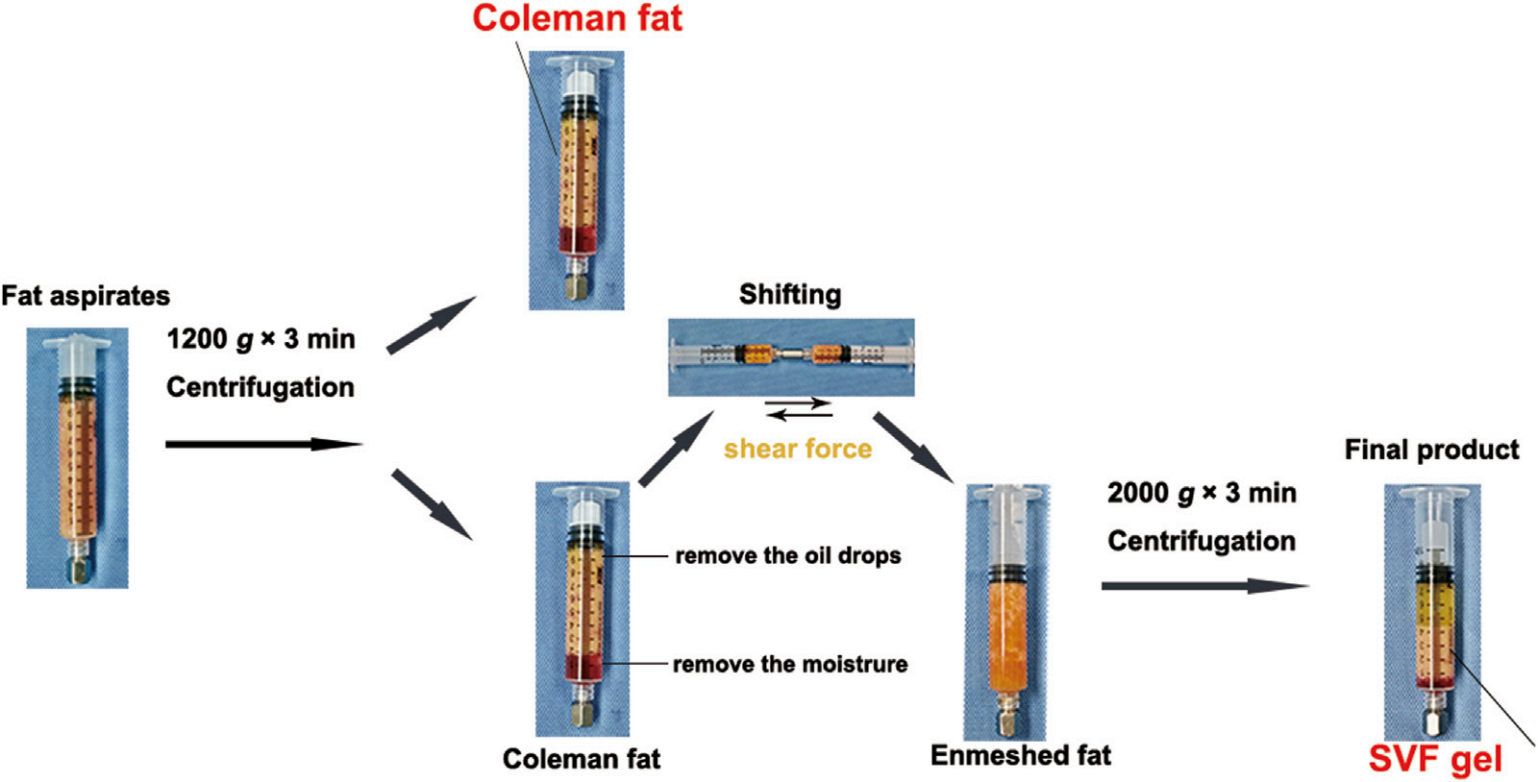
Preparation protocol for stromal vascular fraction (SVF) gel and Coleman fat. Recovery of Coleman fat was achieved by centrifuging the fat aspirates. The SVF gel was obtained by selectively retaining the upper layers containing oil and fat, removing the lower aqueous layer, and subsequently applying pressure to establish the enmeshed fat. This procedure was followed by an additional centrifugation step. This preparation involves the utilization of purely manual techniques, without any dependence on external substances.

To eliminate potential confounding factors such as estrogen (Bills et al., 2015; Ye et al., 2017), we collected abdominal fat samples from ten women undergoing liposuction who did not have any metabolic diseases. The average age and body mass index of these patients were 33.0 (±2.8) years and 20.5 (±1.2) kg/m^2^, respectively. This study was approved by the Medical Ethics Committee of the Guangdong Women and Children’s Hospital (no. 202301333). All participants signed consent documents. The collected fat samples were divided into the Coleman fat group and SVF gel group. Briefly, Coleman fat served as the control group and was extracted using negative-pressure suction into a 10 mL syringe connected to a liposuction needle (Coleman, 2001; Mok et al., 2018). The 10 mL of aspirated fat was centrifuged at 1,200 *g* for 3 min to remove the upper layer of oil and retain the middle layer as Coleman fat. This fat was used as the control group without any further processing. The SVF gel was prepared following the method described by Yao et al. (Yao et al., 2017). Load approximately 8 mL of Coleman fat, obtained through centrifugation, into a 10 mL syringe and connect it to another empty 10 mL syringe using a female-to-female Luer lock connector with an inner diameter of 2.4 mm. Perform reciprocal injection and repeated fragmentation of the adipose tissue. Subsequently, the resulting intertwined adipose tissue was then centrifuged at 2000 *g* for 3 min. The gel-like substance derived from the central portion of the fat tissue, known as SVF gel, was utilized as the experimental group (Figure 1).

### 2.2 Fluid shear force

To compare the shear stress, which was derived from mechanical isolation, between the Coleman fat and SVF gel groups, we measured their respective viscosities. The DV-IPRIME Viscometer (Brookfield Engineering Laboratories, Inc., Boulevard, MA, United States) was used to determine the kinematic viscosity before and after emulsification. In the initial stage, we utilized the equation Re = 2q/π ν R to compute the Reynolds number (Re). Here, q represents the volumetric flow rate of the substance being studied, ν indicates its dynamic viscosity, and R signifies the diameter of the adapter pipe connected to the syringe. If our analysis revealed laminar flow, we determined the shear stress (τ) for fat samples using τ = 4 μ Q/π R3. The dynamic viscosity (μ) was calculated as ν × ρ, with ρ approximating the density of water (Banyard et al., 2016).

### 2.3 Establishment of animal models and formation of experimental groups

Male BALB/C nude mice were chosen as recipients of the fat transplant. The mice were housed, bred, and maintained at the Experimental Animal Center affiliated with Guangzhou Medical University. Ethical approval for conducting animal experiments (reference number GY 2018-018) was obtained from the institutional review board, in accordance with national regulations governing research involving animals. Nude mice (24) were selected for the transplantation of two distinct adipose tissue samples, namely, Coleman fat and SVF gel. Each mouse received 0.1 mL of fat, with one type of fat transplanted onto the left side and the other onto the right side of their backs, injected subcutaneously using a 1 mL syringe. Subcutaneous tissue samples were harvested 0, 3, 28, and 60 days after transplantation. Six mice were randomly chosen for tissue harvesting at each time point. The harvested tissues were divided into two parts: one part was used for histological observations and immunostaining, and the other part was used for microarray analysis and RNA-seq.

### 2.4 Overall appearance and histological observation of fat grafts

All mice were euthanized via intraperitoneal injection of pentobarbital at a dosage of 50 mg/kg. Following transplantation, the fat samples were examined for general and histological changes on days 3, 28, and 60. Subsequently, the long-term retention rates were evaluated. The fat graft samples were dissected and prepared for hematoxylin and eosin (HE) staining and immunofluorescence staining. Immunostaining involved the use of guinea pig anti-mouse perilipin (Progen, Heidelberg, Germany) at a dilution of 1:400 to label mature adipocytes (Doi et al., 2015; Kato et al., 2014; Ye et al., 2017). The Alexa Fluor 647 secondary antibody was labeled with goat anti-guinea pig immunoglobulin G (Abcam, Cambridge, UK) in a dilution ratio of 1:2000. Additionally, nuclear staining was performed using DAPI (Sigma, St. Louis, MO, United States) at a dilution ratio of 1:2000. All data were analyzed independently by two observers.

### 2.5 Flow cytometry for mesenchymal stem cell quantification

The method for preparing the SVF cell suspension from adipose tissue was adopted based on established experimental protocols outlined in previous scholarly literature (Yao et al., 2017). However, at the cell processing stage, we opted to grind and subsequently filter through a 70 μm mesh, instead of using collagenase digestion. This decision was based on our concern that collagenase digestion could potentially impact cell counting and consequently affect experimental outcomes. The expression profile of CD45^−^CD34^+^ serves as a reliable marker for identifying mesenchymal stem cells (Banyard et al., 2016). The surface markers of mesenchymal stem cells within the SVF cell suspension were examined using fluorescence-activated cell sorting technology. This investigation involved the use of specific monoclonal antibodies, including mouse anti-human CD45-APC (BD Pharmingen, California, United States) and CD34-PerCP (Biolegend, California, United States). To identify cell surface markers in the SVF suspension, an LSR II flow cytometer (BD Biosciences) was used.

### 2.6 Global gene expression profiling

After 3 days of fat transplantation, we randomly selected three mice (out of a total of six) and extracted Coleman fat and SVF gel from the dorsal region of each mouse for subsequent global gene expression profiling analysis. The RNA was extracted from adipose tissue graft samples using RNAiso Plus (Takara, Dalian, China), and its integrity was assessed by electrophoretic analysis. The concentration of RNA was quantified using a NanoDrop 2000 spectrophotometer (Thermo Fisher Scientific, Shanghai, China).

To conduct microarray analysis, we performed reverse transcription on the total RNA to generate single-stranded cDNAs using oligo-dT. These cDNAs were transcribed into antisense complementary RNA (cRNA). We employed the Two-Color Agilent Low Input Quick Amp Labeling Kit, provided by Agilent Technologies (Santa Clara, CA, United States), to proficiently label cRNA derived from three mice’s Coleman fat as a universal reference sample using Cy3-CTP. The experimental samples were labeled with Cy5-CTP. Following this labeling step, we fragmented the cRNAs and hybridized them onto Sure Print G3 Human GE 8 × 60 K v2 Microarray Chips, also provided by Agilent Technologies. After completing hybridization and washing procedures, a fluorescence scanner from Agilent was used to capture intensity measurements at wavelengths of 635 nm for Cy5 and 532 nm for Cy3. The resulting image data were acquired using the Agilent Feature Extraction Software. Spots with low intensity were excluded, while ensuring that at least 80% of signals exhibited a sample level above 800 for each gene.

RNA-seq analysis was performed by OE Biotech Co., Ltd. (Shanghai, China). The sequencing libraries were prepared and processed using an Illumina Novaseq 6,000 platform, resulting in the generation of paired-end reads with a length of 150 base pairs. After filtering out low-quality reads, the clean reads were aligned to the reference genomes of humans (GRCh38) and mice (mm9). Fragments per kilobase of transcript per million mapped read (FPKM) values were computed for individual genes and HTSeq-count software was used to obtain gene-specific read counts. Differentially expressed genes (DEGs) meeting the criteria of fold-change >2.0 and q-value <0.05 were identified using SAM. To classify DEGs into distinct biological processes and categories based on Gene Ontology (GO), functional enrichment analysis was performed using R package clusterProfiler v3.2.0 (Roberts et al., 2011).

### 2.7 Statistical analysis

Statistical analysis was performed utilizing SPSS 20.0. Data are expressed as means ± standard deviation. The independent-samples *t*-test was used to compare data between groups at specific time points, with statistical significance defined as *p* < 0.05.

## 3 Results

### 3.1 Correlation between the viscosity of adipose tissue and shear force

**FIGURE 2:**
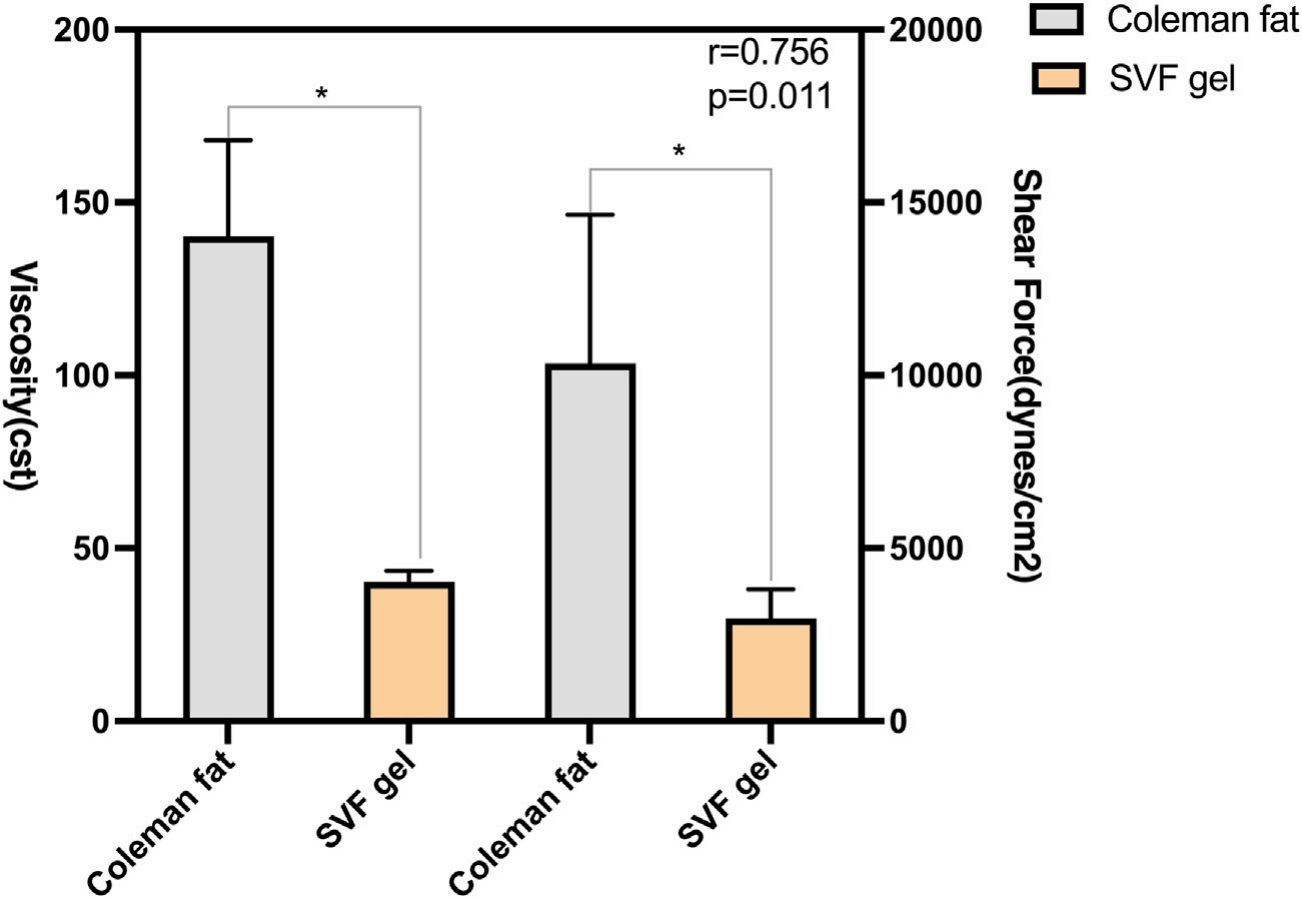
Correlation between the shear force and kinematic viscosity of various types of fats. The SVF gel was obtained from the initial Coleman fat by subjecting it to shear force during mechanical isolation. The viscosity of the fat is positively correlated with shear force, and there is a significant linear relationship between the two.

We investigated the correlation between shear force and kinematic viscosity by conducting mechanical separation of standard Coleman fat into SVF gel (Figure 2). Prior to centrifugation, it was observed that the fat exhibited entanglement, resulting in a significantly higher kinematic viscosity than the SVF gel under identical conditions of connector radius (1.2 mm) and volume flow (10 mL/s). The SVF gel and Coleman fat exhibited laminar flow characteristics, with respective Reynolds numbers of 65.9 and 18.9. The shear force exerted by Coleman fat surpassed that of the SVF gel. The correlation coefficient between viscosity and shear force was equal to 0.756 (*p* = 0.011). These findings suggest a proportional reduction in shear force production capability with decreasing viscosity. Moreover, a strong linear correlation was observed.

### 3.2 Overall observations and enhanced long-term preservation of grafts

**FIGURE 3:**
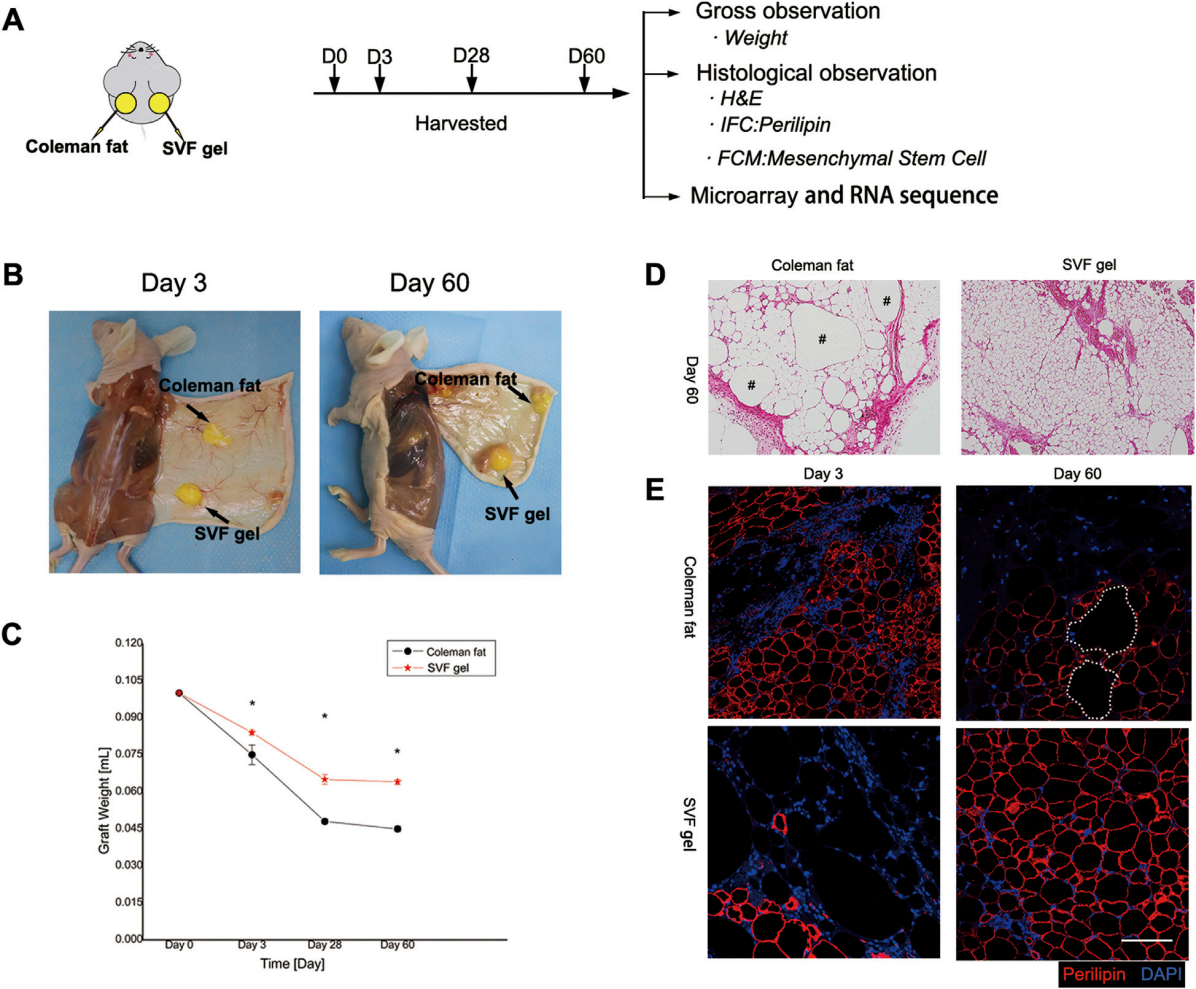
General appearance, longevity, and histological alterations in transplanted adipose tissue. **(A)** Methodology used for experimentation. **(B)** Comprehensive observations conducted on both categories of adipose grafts at post-transplantation intervals of 3 and 60 days. **(C)** Alterations in the rate of adipose tissue preservation. Compared to Coleman fat, SVF gel has a higher retention rate. *p < 0.001. **(D)** Histological analysis conducted 60 days post-transplantation revealed the complete maturation of an organized adipose structure within the SVF gel on the right side, whereas residual oil droplets were observed in the Coleman fat grafts on the left side. The scale bar used for microscopic examination was set at 200 μm. The symbol “#” denotes the presence of oil droplets. **(E)** Immunofluorescence staining demonstrated that 3 days after transplantation, necrotic signs appeared among adipocytes within SVF gel grafts along with a significant increase in areas negative for perilipin staining compared with Coleman fat grafts. This suggests that mature adipocytes within the SVF gel were subjected to shear force-induced damage. However, after 60 days of transplantation, numerous newly formed adipocytes with positive perilipin staining were observed in the SVF gel grafts on the lower right side, whereas multiple regions lacking perilipin staining were detected in the Coleman fat grafts on the upper right side. These findings indicate a higher incidence of adipocyte neogenesis within the SVF gel and more instances of adipocyte necrosis within Coleman fat. The scale bars used for microscopic examination were set at 100 μm. Statistical analysis indicated significance at *p* < 0.001.

Samples should be collected at various time points to facilitate a comparative analysis of the differences in retention rate, histological characteristics, and cellular molecular biology aspects between the two types of adipose tissue (Figure 3A). The SVF gel group demonstrated significantly higher retention rates compared to the Coleman fat group at 3, 28, and 60 days post-transplantation (0.084 ± 0.001 vs. 0.075 ± 0.004; 0.065 ± 0.002 vs. 0.048 ± 0.001; and 0.064 ± 0.001 vs. 0.045 ± 0.001; *p* < 0.001 for all time points; Figures 3B, C). These findings suggest that the SVF gel group exhibits superior retention effects over the Coleman fat group at different time points.

### 3.3 Histological observation of grafts

On the 60th day post-transplantation, histological analysis using hematoxylin and eosin staining revealed a well-developed and organized adipose structure in the stromal vascular fraction (SVF) gel group. In contrast, the Coleman fat group exhibited numerous lipid droplets derived from necrotic adipocytes, leading to an immature adipose structure (Figure 3D). On the third day post-transplantation, a significant reduction in mature adipocyte count was observed in the SVF gel group compared to the Coleman fat graft group, as indicated by absence of perilipin staining. Interestingly, by day 60 after transplantation, extensive areas with negative perilipin staining were evident within the Coleman fat group, suggesting occurrence of adipocyte necrosis (Figure 3E). In SVF gel, early mature adipocytes undergo apoptosis, whereas late-stage ones exhibit the formation of fully organized adipose structures. This phenomenon is attributed to the application of mechanical shear force and the subsequent differentiation of a large number of regenerative cells into adipocytes.

### 3.4 Mesenchymal stem cell detection

**FIGURE 4:**
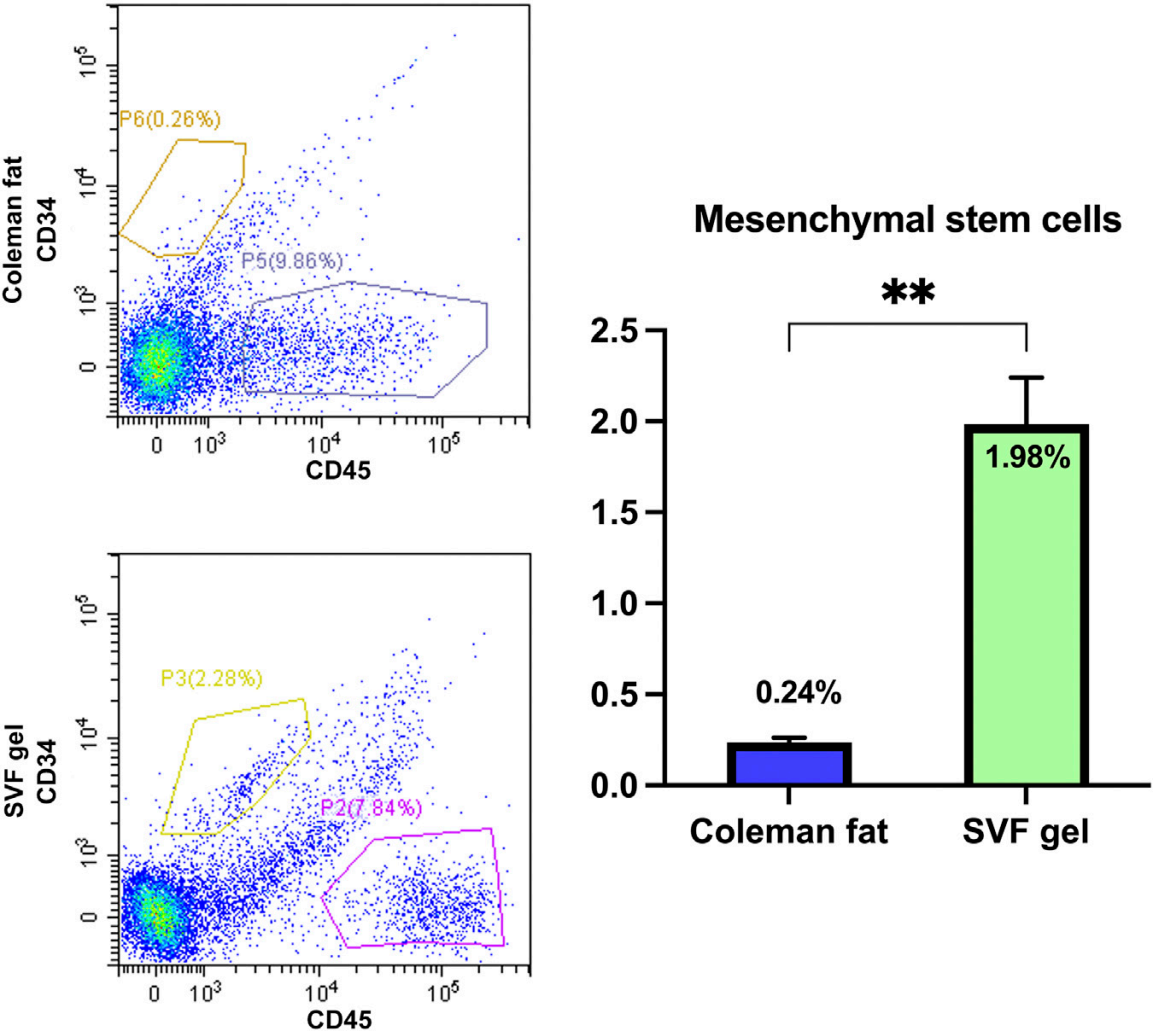
Quantification of the relative abundance of mesenchymal stem cells in various tissues using flow cytometry. The flow cytometric analysis findings revealed a significant increase in the abundance of mesenchymal stem cells within the SVF gel compared with that observed in Coleman fat, with a difference exceeding eightfold. **p < 0.01.

The CD34^+^/CD45^-^ cells were identified as mesenchymal stem cells (MSCs) (Banyard et al., 2016). Compared to Coleman fat, the number of CD34^+^/CD45^-^ cells in SVF gel exhibited a significant increase by more than 8-fold (1.98 ± 0.258 vs. 0.24 ± 0.025; *p* < 0.01). (The flow cytometry data is included in the Supplementary Material S1). The utilization of mechanical shear force during the process of SVF gel preparation significantly enhanced the proportion of mesenchymal stem cells, indicating its potential to induce phenotypic changes and promote an increase in regenerative-related cell populations (Figure 4).

### 3.5 Validating the origin of newly generated adipose tissue post-transplantation using transcriptomic techniques

**FIGURE 5:**
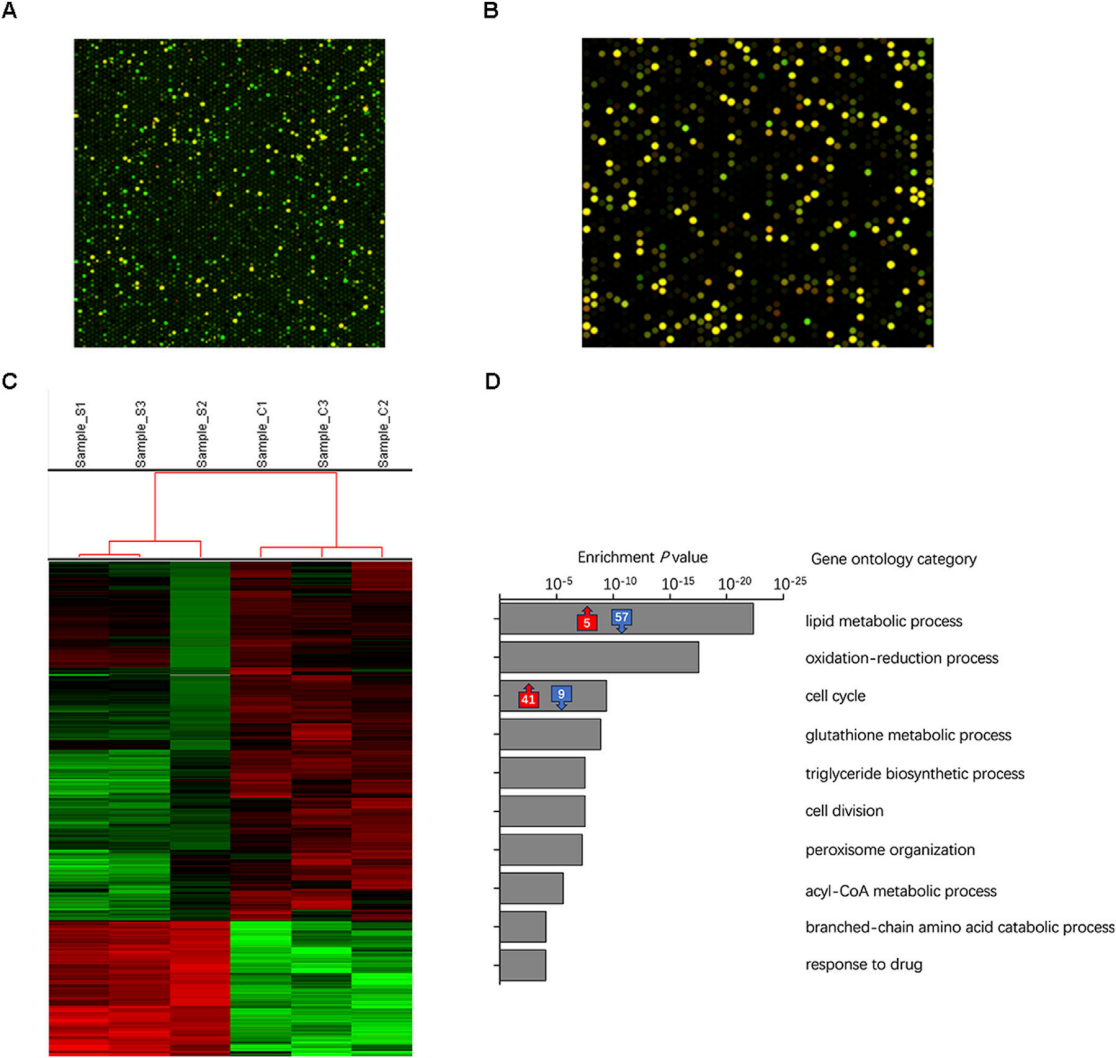
Identification of tissue origin during early stages of transplantation was performed using microarray and RNA-seq analysis on transplanted tissues derived from the SVF gel and Coleman fat groups. **(A)** Dot-matrix graphics of the human cDNA microarray of transplanted adipose tissue on day three show the low quality of the hybridization signal. This schematic diagram is based on samples obtained from the SVF gel group in this study. **(B)** High-quality hybridization signal for human tissue directly analyzed following liposuction after background correction. The schematic sample is a recently acquired specimen of Coleman fat that has not undergone any transplantation treatment. **(C)** The heatmap illustrates the expression levels of individual genes, with colors ranging from green to red indicating low to high levels. **(D)** The gray box on the right displays the top 10 significantly enriched biological processes/categories, along with the corresponding number of upregulated/downregulated expressed genes involved in lipid metabolism and cell cycle processes.

Although an adequate amount of RNA was extracted from the adipose tissue of the transplant sites after 3 days, the hybridization signal between the transplanted fat sample and the human microarray chip was generally low (Figure 5A). The median value of the red and green signals of the transplanted fat after background correction was approximately 10, whereas that of a control sample obtained directly after liposuction was approximately 50 (Figure 5B). After eliminating weak signals, the number of expressed genes significantly decreased, rendering subsequent differential gene expression analysis ineffective. These findings suggested that the primary source of generated adipocytes originated from the host mice by the third day after transplantation, with only a minimal presence of human-derived cells.

We utilized RNA-seq to analyze the transcriptome of the adipose tissues to simultaneously examine gene expression in mice and humans. The proportion of genes expressed from human sources was considerably smaller than that expressed from mouse sources in both the Coleman fat and SVF gel groups, which correlated with the microarray results. When focusing on human-expressed genes for analysis, only three DEGs were identified. These results indicated that the primary cells in the implanted adipose tissue became inactive by the third day after implantation, whereas the newly formed tissue became the predominant component (Chen et al., 2024).

### 3.6 Distinct biological processes in Coleman fat and SVF gel

We conducted an analysis of the distinct functional categories during the initial stages following SVF gel implantation, which was prepared using mechanical force based on RNA-seq data. Figure 5C presents a heatmap showcasing DEGs observed in regenerated tissues derived from mice. The comparison between the SVF gel group and the Coleman fat group revealed 601 differentially expressed genes (DEGs). Among them, 164 were upregulated, whereas 437 were downregulated. The top 10 biological processes exhibiting significant enrichment are presented (Figure 5D). The GO enrichment analysis indicated that the DEGs primarily corresponded to two key biological processes: lipid metabolism and the cell cycle process. Within the lipid metabolism process, 62 DEGs were enriched, with 57 downregulated and five upregulated in the SVF gel group; the five upregulated genes were associated with negative regulation of lipid metabolism. This suggested that the Coleman group displayed highly active lipid metabolism compared with the SVF gel group. In contrast, 50 DEGs were significantly enriched within the cell cycle, with 41 upregulated and nine downregulated. These nine downregulated genes were also identified as negative regulators of cell division. The Supplementary Material comprises the primary enriched GO terms and an exhaustive list of DEGs.

## 4 Discussion

In the past decade, there has been a notable transition in the concept of fat regeneration, moving away from the initial theory focused on graft survival towards an emphasis on the theory of tissue rejuvenation (Coleman, 2006a; Doi et al., 2015; Eto et al., 2012; Yuan et al., 2015). Researchers have actively worked on improving adipose tissue regeneration after transplantation by developing new techniques for fat preparation and processing (Canizares et al., 2017; Spyropoulou, 2022; Streit et al., 2017; Vyas et al., 2020). Currently, the strategy to improve the viability of fat grafts focuses on augmenting the number of cells associated with regeneration, including the SVF and ASCs (Bashir et al., 2019; Cai et al., 2018; Kim et al., 2019).

Host-origin cells are now widely accepted to populate the graft (Doi et al., 2015; Tang et al., 2018; Yao et al., 2017). However, the proportion of recipient and donor cells within the graft and the molecular mechanisms underlying the improvement of fat retention and adipose tissue regeneration with SVF/enzyme-digested collagen matrix (ECM) gel compared with traditional Coleman fat remain unclear. To address this, we performed microarray analysis to determine the cell sources within grafts derived from human fat that were directly analyzed after liposuction and then transplanted onto the backs of mice on day three. Our findings revealed that the cells within the grafts were primarily of murine origin, with very few detectable human-derived cells. These results align with those of previous studies (Kato et al., 2014; Yao et al., 2017) that utilized double immunofluorescence analysis, labeling green fluorescent protein and perilipin to confirm the origin of regenerated adipocytes. However, our study provides novel insights through a comprehensive analysis of cellular outcomes in adipose tissue post-transplantation using molecular biology techniques, which serves as a valuable supplement to previous findings (Kato et al., 2014; Yao et al., 2017).

Our previous investigation observed that Coleman fat exhibited a relatively higher count of viable cells without mechanical separation. Conversely, the application of shear force resulted in an increased distribution ratio of distinct cell subgroups within the SVF mixed-cell population residing within the adipose tissue (Ye et al., 2021). This led us to question whether the rise in the relative ratio of cells was due to a reduction in non-regeneration-related cell count or an actual augmentation in the quantity of regeneration-related cells. Consequently, the aim of the present study was to elucidate the molecular mechanism underlying shear force-induced regeneration at the transcriptomic level using RNA-seq. From the third day post-transplantation, we observed an upregulation of genes associated with cell division signaling pathways and a downregulation of genes involved in lipid metabolism signaling pathways in the SVF gel group compared with the Coleman fat group. This indicated that cell division and proliferation were highly activated in the SVF gel group. This observation suggests a robust activation of cell division and proliferation in the SVF gel group, indicating heightened cellular activity.

A considerable number of mature fat cells were retained in Coleman fat. However, these mature adipocytes experienced hypoxia and nutrient limitations after transplantation. Consequently, many central adipocytes underwent cell death, releasing oil droplets and initiating an intense lipid metabolism process (Kato et al., 2014). Conversely, mature adipocytes were disrupted using mechanical shear force in the process of SVF gel production, followed by subsequent removal of oil droplets through centrifugation (Sesé et al., 2019; Spyropoulou, 2022). Consequently, the lipid metabolism signaling pathway was downregulated compared with Coleman fat, potentially attenuating inflammatory responses and reducing the risk of postoperative complications in adipose tissue grafts. Concurrently, the upregulation of genes associated with the cellular mitosis signaling pathway may increase the relative abundance of cells involved in regeneration. Thus, we hypothesize that the increase in cell division and proliferation may lead to an absolute increase in the number of regenerated cells. These two findings suggest potential advantages of clinically utilizing SVF gel over Coleman fat in transplantation scenarios.

Mechanical shear forces enhance pluripotency and provide a rich source of regenerative cells for cellular therapy (Zhou, et al., 2024a). These forces exert a positive influence on signaling pathways associated with cell proliferation and differentiation (Zhou, et al., 2024b). Based on the transcriptome results, we will conduct further experimental investigations to elucidate the influence of mechanical treatment on adipose tissue transplantation by focusing on its impact on cell proliferation pathways.

We are studying the limitations of not elucidating the key genes in the signaling pathway. In the future, we will clarify these key genes and further experimentally validate them, with the aim of upregulating or downregulating these key genes to improve transplant fat retention rates and reduce complications.

Our study provides evidence that physical effects applied to fat tissue can translate into biological effects post-transplantation. Mechanical shear force effectively induces adipose regeneration in the recipient by upregulating the cellular mitosis signaling pathway and simultaneously downregulating the lipid metabolism signaling pathway. Importantly, this study further verified the mode of fat regeneration after transplantation at the molecular level. This knowledge serves as a theoretical foundation for achieving efficient and stable tissue augmentation, thereby promoting future advancements in clinical tissue repair and reconstruction.

## Data Availability

The datasets presented in this study can be found in online repositories. The names of the repository/repositories and accession number(s) can be found in the article/Supplementary Material.
