# Supplementary figures and images for: Mechanical force promotes tissue and molecular changes in adipose tissue regeneration post-transplantation
Source: Front Cell Dev Biol. 2024 Sep 18;12:1472575. doi: 10.3389/fcell.2024.1472575 (PMC11445162; doi:10.3389/fcell.2024.1472575)

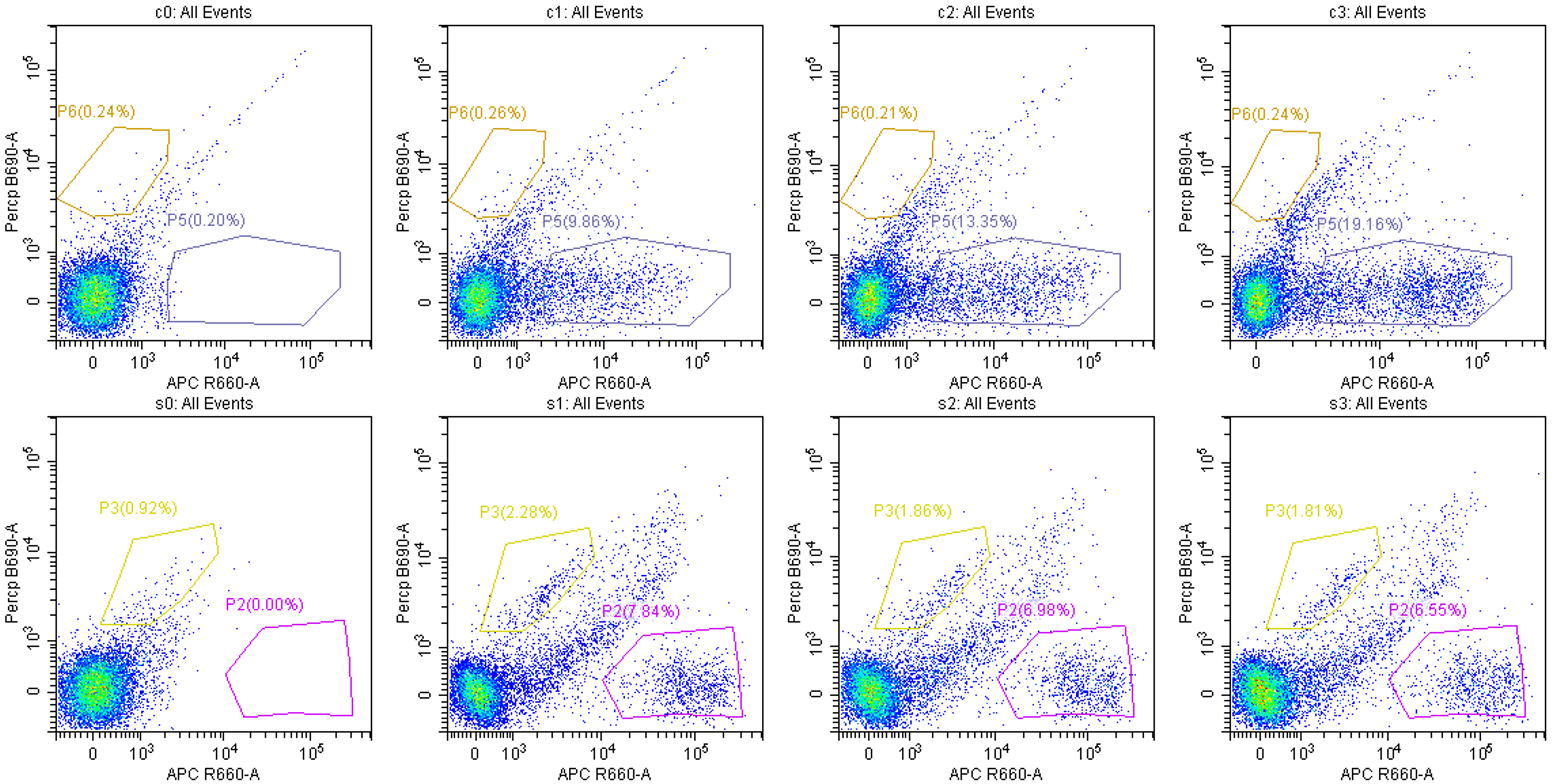

Supplement: Supplementary file 1 [file Image1.tif]
